# Supplementary material for: Combating pancreatic cancer with ovarian cancer cells
Source: Aging (Albany NY). 2023 Mar 23;15(6):2189–207. doi: 10.18632/aging.204608 (PMC10085619; doi:10.18632/aging.204608)
Supplement: Supplementary Files 1-3 [file aging-15-204608-s001.pdf]

SUPPLEMENTARY FILES

Supplementary File 1. Comparison of the tumor volume between treatment group and NSCT group during intervention.

Tumor volume	One way ANOVA
data 1	
day1	P-value
Gemcitabine vs. NSCT	0.886
SKOV3 HD vs. NSCT	0.755
SKOV3 LD vs. NSCT	0.591
day4	
Gemcitabine vs. NSCT	0.00108
SKOV3 HD vs. NSCT	0.00068
SKOV3 LD vs. NSCT	0.0012
day7	
Gemcitabine vs. NSCT	0.00028
SKOV3 HD vs. NSCT	0.00276
SKOV3 LD vs. NSCT	0.00747
day11	
Gemcitabine vs. NSCT	0.00221
SKOV3 HD vs. NSCT	0.00978
SKOV3 LD vs. NSCT	0.02112
data 2	
day1	
CT	
Gemcitabine vs. NSCT	0.988723910927881
$SKOV3^{GFP}$ HD vs. NSCT	0.799224687648322
$SKOV3^{GFP}$ LD vs. NSCT	0.581036672963136
$SKOV3^{GFP+TP53}$ HD vs. NSCT	0.192442062966073
$SKOV3^{GFP+TP53}$ LD vs. NSCT	0.321056162570084
day3	
Gemcitabine vs. NSCT	0.0489434728145107
$SKOV3^{GFP}$ HD vs. NSCT	0.00633237051383828
$SKOV3^{GFP}$ LD vs. NSCT	0.0124801477653647
$SKOV3^{GFP+TP53}$ HD vs. NSCT	0.0147437002104617
$SKOV3^{GFP+TP53}$ LD vs. NSCT	0.00670137653176828
day5	
Gemcitabine vs. NSCT	6.83945773338746E-06
$SKOV3^{GFP}$ HD vs. NSCT	0.000231533672790163
$SKOV3^{GFP}$ LD vs. NSCT	0.000371949680032069
$SKOV3^{GFP+TP53}$ HD vs. NSCT	0.0000435305961362653
$SKOV3^{GFP+TP53}$ LD vs. NSCT	5.84198733122321E-06

day8	
Gemcitabine vs. NSCT	1.46568033265737E-08
SKOV3^{GFP} HD vs. NSCT	6.18821703324279E-07
SKOV3^{GFP} LD vs. NSCT	5.66388905776094E-07
SKOV3$^{GFP+TP53}$ HD vs. NSCT	1.45506490778491E-06
SKOV3$^{GFP+TP53}$ LD vs. NSCT	2.78109778428792E-07
day10	
CT	
Gemcitabine vs. NSCT	8.74263457739666E-07
SKOV3^{GFP} HD vs. NSCT	0.0000127015491199201
SKOV3^{GFP} LD vs. NSCT	0.0000178679134628101
SKOV3$^{GFP+TP53}$ HD vs. NSCT	0.000558596974845615
SKOV3$^{GFP+TP53}$ LD vs. NSCT	0.0000569740943069386
day12	
Gemcitabine vs. NSCT	0.0000419058969515218
SKOV3^{GFP} HD vs. NSCT	0.000235004033309782
SKOV3^{GFP} LD vs. NSCT	0.000118407204488857
SKOV3$^{GFP+TP53}$ HD vs. NSCT	0.00741653187530478
SKOV3$^{GFP+TP53}$ LD vs. NSCT	0.000211811185322874
day14	
Gemcitabine vs. NSCT	0.0000908113295305935
SKOV3^{GFP} HD vs. NSCT	0.000421649926519496
SKOV3^{GFP} LD vs. NSCT	0.00101226327008364
SKOV3$^{GFP+TP53}$ HD vs. NSCT	0.00527982130533911
SKOV3$^{GFP+TP53}$ LD vs. NSCT	0.000241841375546059
day16	
Gemcitabine vs. NSCT	0.0000850007428913058
SKOV3^{GFP} HD vs. NSCT	0.00020203430765342
SKOV3^{GFP} LD vs. NSCT	0.00034785890476996
SKOV3$^{GFP+TP53}$ HD vs. NSCT	0.00164519610173053
SKOV3$^{GFP+TP53}$ LD vs. NSCT	0.000394975002415032
day18	
Gemcitabine vs. NSCT	0.0000168279512249292
SKOV3^{GFP} HD vs. NSCT	0.0000222563302877399
SKOV3^{GFP} LD vs. NSCT	0.00267032142927348
SKOV3$^{GFP+TP53}$ HD vs. NSCT	0.000443752799526576
SKOV3$^{GFP+TP53}$ LD vs. NSCT	0.000133607047711926

Supplementary File 2. The enriched disease terms of DEGs between the tumors with and without injection of ovarian cancer cells.

Category	Term	FDR
ClinVar	hereditary pancreatitis	0.0000000000000317
DisGeNET	Autosomal Dominant Hereditary Pancreatitis	0.000000000000349
DisGeNET	Abnormal enzyme/coenzyme activity	0.0000000000323
DisGeNET	Pancreatitis, Alcoholic	0.00000000314
DisGeNET	Elevated C-reactive protein	0.00000000646
DisGeNET	Elevated C-reactive protein level	0.00000000646
DisGeNET	Leukocytosis	0.0000000502
DisGeNET	Collagenopathy, type 2 alpha 1	0.00000338
DisGeNET	Pancreatic disorders (not diabetes)	0.00000789
DisGeNET	Pancreatitis, Chronic	0.0000565
DisGeNET	Hereditary pancreatitis	0.0000956
DisGeNET	Gastrointestinal pain	0.000101
DisGeNET	Bacterial sepsis of newborn	0.000207
DisGeNET	Pancreatic Insufficiency	0.000302
DisGeNET	Abdominal discomfort	0.000302
DisGeNET	Sepsis of the newborn	0.000351
DisGeNET	Exocrine pancreatic insufficiency	0.000538
DisGeNET	Abdominal Pain	0.00107
DisGeNET	Malignant tumor of eye	0.00171
DisGeNET	Pancreatic Pseudocyst	0.00563
DisGeNET	Bronchopulmonary Dysplasia	0.00747
DisGeNET	Hypoproteinemia	0.00777
DisGeNET	Idiopathic chronic pancreatitis	0.00777
DisGeNET	Intrahepatic Cholangiocarcinoma	0.00777
DisGeNET	Axenfeld-Rieger syndrome	0.00849
DisGeNET	Abnormal thrombosis	0.0117
DisGeNET	Carcinoma, Signet Ring Cell	0.0122
DisGeNET	TROPICAL CALCIFIC PANCREATITIS	0.0133
DisGeNET	Pancreatic Ductal Adenocarcinoma	0.0154
DisGeNET	Ki-1+ Anaplastic Large Cell Lymphoma	0.0189
DisGeNET	Colorectal Neoplasms	0.021
DisGeNET	Acute recurrent pancreatitis	0.021
DisGeNET	Autoimmune pancreatitis	0.0234
DisGeNET	Perisylvian syndrome	0.0278
DisGeNET	Age related macular degeneration	0.0311
DisGeNET	Pediatric Crohn's disease	0.0413
GWAS Catalog 2019	Pancreatitis	7.41E-19
GWAS Catalog 2019	Blood protein levels	0.000101
GWAS Catalog 2019	Methadone dose in opioid dependence	0.0433
GWAS Catalog 2019	Cancer	0.0454
Human_Phenotype_Ontology	Hypoproteinemia (HP:0003075)	0.000357
Human_Phenotype_Ontology	Pancreatitis (HP:0001733)	0.000565

Human_Phenotype_Ontology	Steatorrhea (HP:0002570)	0.0179
Human_Phenotype_Ontology	Exocrine pancreatic insufficiency (HP:0001738)	0.0186
Human_Phenotype_Ontology	Abnormality of the pleura (HP:0002103)	0.0221
Human_Phenotype_Ontology	Anal atresia (HP:0002023)	0.0254
Human_Phenotype_Ontology	Fat malabsorption (HP:0002630)	0.0289
Jensen_DISEASES	Pancreatic steatorrhea	0.000114
Jensen_DISEASES	Steatorrhea	0.000438
Jensen_DISEASES	Shipyard eye	0.00139
Jensen_DISEASES	Pancreatitis	0.00139
Jensen_DISEASES	Pneumothorax	0.031
Rare_Diseases	Hereditary pancreatitis	0.0000369
Rare_Diseases	Pancreatitis pediatric	0.000843
Rare_Diseases	Pediatric ulcerative colitis	0.00342

Supplementary File 3. The enriched kinases of DEGs.

Term	FDR
PRKCG human kinase ARCHS4 coexpression	0.00000202
MAST1 human kinase ARCHS4 coexpression	0.00000995
CAMKV human kinase ARCHS4 coexpression	0.00000995
STK32B human kinase ARCHS4 coexpression	0.0000122
LMTK3 human kinase ARCHS4 coexpression	0.0000153
TTBK1 human kinase ARCHS4 coexpression	0.0000153
CAMK1G human kinase ARCHS4 coexpression	0.0000153
CAMK2B human kinase ARCHS4 coexpression	0.0000317
CAMK1D human kinase ARCHS4 coexpression	0.0000317
MAPK15 human kinase ARCHS4 coexpression	0.0000317
ANKK1 human kinase ARCHS4 coexpression	0.0000317
NEK10 human kinase ARCHS4 coexpression	0.0000317
ALPK2 human kinase ARCHS4 coexpression	0.000078
BRSK2 human kinase ARCHS4 coexpression	0.000078
MAPK4 human kinase ARCHS4 coexpression	0.000078
MAP3K9 human kinase ARCHS4 coexpression	0.000078
MYLK human kinase ARCHS4 coexpression	0.000168
DDR2 human kinase ARCHS4 coexpression	0.000168
CAMK2G human kinase ARCHS4 coexpression	0.000168
PRKCZ human kinase ARCHS4 coexpression	0.000168
PAK1 human kinase ARCHS4 coexpression	0.000168
SBK1 human kinase ARCHS4 coexpression	0.000168
MAP3K19 human kinase ARCHS4 coexpression	0.000168
NEK11 human kinase ARCHS4 coexpression	0.000168
NEK7 human kinase ARCHS4 coexpression	0.000389
WNK2 human kinase ARCHS4 coexpression	0.000389
ERN2 human kinase ARCHS4 coexpression	0.000389
BRSK1 human kinase ARCHS4 coexpression	0.000389
PNCK human kinase ARCHS4 coexpression	0.000389
DCLK2 human kinase ARCHS4 coexpression	0.000389
PAK6 human kinase ARCHS4 coexpression	0.000389
PLK5 human kinase ARCHS4 coexpression	0.00084
PDGFRB human kinase ARCHS4 coexpression	0.000869
PDGFRA human kinase ARCHS4 coexpression	0.000869
FLT1 human kinase ARCHS4 coexpression	0.000869
CAMK2A human kinase ARCHS4 coexpression	0.000869
PRKCE human kinase ARCHS4 coexpression	0.000869
CDK15 human kinase ARCHS4 coexpression	0.000869
TYRO3 human kinase ARCHS4 coexpression	0.000869
CAMK4 human kinase ARCHS4 coexpression	0.000869
KALRN human kinase ARCHS4 coexpression	0.000869
MAPK11 human kinase ARCHS4 coexpression	0.00172
TEK human kinase ARCHS4 coexpression	0.00205

MAP3K10 human kinase ARCHS4 coexpression	0.00205
CAMKK1 human kinase ARCHS4 coexpression	0.00205
PAK5 human kinase ARCHS4 coexpression	0.00205
MAK human kinase ARCHS4 coexpression	0.00205
EPHA6 human kinase ARCHS4 coexpression	0.00205
MOK human kinase ARCHS4 coexpression	0.00205
GRK7 human kinase ARCHS4 coexpression	0.0027
ACVR1 human kinase ARCHS4 coexpression	0.00455
BLK human kinase ARCHS4 coexpression	0.00456
CAMKK2 human kinase ARCHS4 coexpression	0.00456
EPHB6 human kinase ARCHS4 coexpression	0.00456
MAP3K12 human kinase ARCHS4 coexpression	0.00456
DCLK1 human kinase ARCHS4 coexpression	0.00456
MAPK10 human kinase ARCHS4 coexpression	0.00456
NRBP2 human kinase ARCHS4 coexpression	0.0084
AATK human kinase ARCHS4 coexpression	0.00861
MAP4K1 human kinase ARCHS4 coexpression	0.0102
BMX human kinase ARCHS4 coexpression	0.0103
MAP2K4 human kinase ARCHS4 coexpression	0.0104
NLK human kinase ARCHS4 coexpression	0.0104
ALK human kinase ARCHS4 coexpression	0.0182
FLT4 human kinase ARCHS4 coexpression	0.0211
FGFR3 human kinase ARCHS4 coexpression	0.0211
ACVRL1 human kinase ARCHS4 coexpression	0.0211
PDK4 human kinase ARCHS4 coexpression	0.0211
FGFR2 human kinase ARCHS4 coexpression	0.0211
BTK human kinase ARCHS4 coexpression	0.0211
HIPK2 human kinase ARCHS4 coexpression	0.0211
NRK human kinase ARCHS4 coexpression	0.0211
CDK14 human kinase ARCHS4 coexpression	0.0212
RPS6KL1 human kinase ARCHS4 coexpression	0.0213
CDK18 human kinase ARCHS4 coexpression	0.0369
RPS6KA2 human kinase ARCHS4 coexpression	0.042
MAP4K2 human kinase ARCHS4 coexpression	0.0426
TIE1 human kinase ARCHS4 coexpression	0.0431
TNNI3K human kinase ARCHS4 coexpression	0.0439
PRKACB human kinase ARCHS4 coexpression	0.0439
NTRK3 human kinase ARCHS4 coexpression	0.0439
