# Supplementary material for: Combating pancreatic cancer with ovarian cancer cells
Source: Aging (Albany NY). 2023 Mar 23;15(6):2189–207. doi: 10.18632/aging.204608 (PMC10085619; doi:10.18632/aging.204608)
Supplement: Supplementary File 5 [file aging-15-204608-s003.pdf]

Supplementary File 5. The significant genes connected with DEGs.

Term	FDR
GDF11	0.000000193
GSTT1	0.0000011
FN1	0.0000011
ITGA4	0.0000011
TUBB1	0.0000284
TUBB3	0.000354
HSPA5	0.000445
